# Could Total Neoadjuvant Therapy Followed by Surgical Resection Be the New Standard of Care in Pancreatic Cancer? A Systematic Review and Meta-Analysis

**DOI:** 10.3390/jcm11030812

**Published:** 2022-02-03

**Authors:** Ottavia De Simoni, Marco Scarpa, Caterina Soldà, Francesca Bergamo, Sara Lonardi, Alberto Fantin, Pierluigi Pilati, Mario Gruppo

**Affiliations:** 1 Unit of Surgical Oncology of Digestive Tract, Veneto Institute of Oncology IOV-IRCCS, 35128 Padua, Italy; ottavia.desimoni@iov.veneto.it (O.D.S.); pierluigi.pilati@iov.veneto.it (P.P.); 2 Department of Surgery, Oncology and Gastroenterology, University Hospital of Padua, 35128 Padova, Italy; marcoscarpa73@yahoo.it; 3 Unit of Medical Oncology 1, Veneto Institute of Oncology IOV-IRCCS, 35128 Padua, Italy; caterina.solda@iov.veneto.it (C.S.); francesca.bergamo@iov.veneto.it (F.B.); 4 Unit of Medical Oncology 3, Veneto Institute of Oncology IOV-IRCCS, 35128 Padua, Italy; sara.lonardi@iov.veneto.it; 5 Department of Gastroenterology, Veneto Institute of Oncology IOV-IRCCS, 35128 Padua, Italy; alberto.fantin@iov.veneto.it

**Keywords:** total neoadjuvant therapy, pancreatic cancer, induction chemotherapy, radio-chemotherapy, pancreatectomy, pancreatic surgery, overall survival

## Abstract

Background. Total neoadjuvant therapy (TNT), intended as induction chemotherapy (IC) followed by radio-chemotherapy (RCT), has been taking hold in the treatment of pancreatic ductal adenocarcinoma (PDAC). The aim of this review is to summarize the available evidence on the role of TNT followed by curative surgery. Methods. Eligible studies were those reporting on patients with PDAC undergoing curative surgery after TNT. The primary endpoint was overall survival (OS). Results. A total of 1080 patients with PDAC who had undergone TNT were analyzed. The most common IC regimen was Gemcitabine (N 620, 57%). Toxicity during IC varied from 14% to 51%. Disease progression during IC varied from 3% to 25%. 607 (62%) patients underwent curative surgery after IC + CRT. In meta-analysis, the available data on lymph node metastases radicality and 2 years OS had better results in favor of TNT groups (OR 1.77, 95% CI 1.20–2.60, *p* = 0.004 and OR 2.03, 95% CI 1.19–3.47, *p* = 0.01 and OR 1.64, CI 1.09–2.47, *p* = 0.02, respectively). Conclusions. Despite the heterogeneity of the studies, different selection criteria, and non-negligible drop-out rate, TNT demonstrated a potential superiority to NAT without CRT in oncological and pathological outcomes, even if the main differences seem to depend on the IC regimen.

## 1. Introduction

Pancreatic ductal adenocarcinoma (PDAC) is one of the leading causes of cancer mortality in developed countries and one of the most lethal malignant neoplasms across the world [1]. Surgical resection in combination with adjuvant systemic chemotherapy is still the standard of care with curative intent [2,3]. However, at diagnosis, only 15–20% of patients are resectable and about 30% have locally advanced unresectable tumors and are generally given palliative measures only [4,5,6].

Neoadjuvant chemotherapy (NAT) is increasingly administered to borderline-resectable (BR) and locally advanced (LA) PDAC with the achievement of a higher percentage of resectability and improvement of oncological outcomes [7,8]. The concept of neoadjuvant rather than adjuvant treatment in PDAC is attractive for several reasons: downstaging of large tumors to allow margin negative resections, facilitate improved patient selection for resection by revealing biological aggressiveness, allow for further observation of indeterminate extrapancreatic lesions prior to resection, and enable medical optimization prior to surgery [9,10,11,12]. Second, up to 30% of the patients cannot receive adjuvant therapy because of poor post-operative performance status [13]. Recently, the concept of total neoadjuvant chemotherapy (TNT) benefiting from both the possible advantages of neoadjuvant therapy and radiotherapy is developing an increasing interest but is not widely accepted [14,15]. The purpose of this systematic review is to summarize the available evidence on the role of TNT followed by surgery with curative intent.

## 2. Materials and Methods

### 2.1. Study Design

We performed a systematic literature search, study design, and data analysis following PRISMA (Preferred Reporting Items for Systematic Reviews and Meta-Analyses) guidelines [16].

### 2.2. Search Strategy

Five medical databases were consulted in this research: Medline, Embase, Cochrane Database of Systematic Reviews, Web of Science, and Scopus. The primary search strategy included keywords and medical subject headings as follows: “Pancreatic cancer,” “Pancreatic cancers,” “Cancer of pancreas,” “Cancer of the pancreas,” “Duct cell carcinoma of the pancreas,” “Ductal carcinoma of the pancreas,” “Total neoadjuvant chemotherapy,” “Total neoadjuvant therapy,” and “Total neoadjuvant treatment.” Articles from the search results have been selected independently by two authors (M.G. and O.D.) following the inclusion and exclusion criteria. Any disagreements in study inclusion between the two authors were resolved by discussion. Only clinical studies written in English were selected. We did not include data quoted as unpublished or derived from abstracts.

### 2.3. Selection Criteria and Outcome Measures

We included all studies investigating a series of patients with a diagnosis of PDAC who underwent TNT followed by curative surgery. We considered TNT a protocol consisting of a phase of induction chemotherapy (IC) followed by a phase of radiotherapy with concurrent chemotherapy (CRT). For the purpose of this review, chemotherapy should be considered as “induction” if it is administered before radiotherapy and “concurrent” if administered during the course of radiotherapy. In the case of duplicate publications that reported on (parts of) similar patient data, only the most recent and complete data sets were considered. Exclusion criteria were as follows: <15 total patients. According to the PICOS criteria, articles were selected in this systematic review according to the follow eligibility criteria: (1) participants: adults with diagnosis of PDAC; (2) intervention: TNT followed by curative surgery; (3) comparison: patients with PDAC undergoing surgery first or neoadjuvant chemotherapy followed by surgery; (4) outcomes: the main outcome measure was overall survival (OS); secondary endpoints were tolerance to TNT, pathological and surgical outcomes, and disease-free survival (DFS).

### 2.4. Data Extraction

Data were extracted from original articles only using a set of predetermined parameters: demographic data, localization of cancer, histological details of PDAC, type of TNT, tolerance to TNT, type of surgery, morbidity, 90 days mortality, DFS, and OS.

### 2.5. Quality Assessment of Retrieved Articles

Two researchers independently assessed the quality of the articles using a quality evaluation list constructed with predefined parameters including: number of patients, accurate description of IC regimens, CRT regimen, surgical procedure, and accurate analysis of response and tolerance to IC + CRT. Moreover, the Newcastle–Ottawa Scale (NOS) was utilized for assessing the quality of non-randomized studies in systematic review analyses.

### 2.6. Statistical Analysis

Review Manager 5.3 (Cochrane Collaboration, Nordic Cochrane Centre, Copenhagen, Denmark) was used for data analysis. All statistical measures were assessed with *p* 0.05 significance level. The I^2^ statistic was used to determine the heterogeneity of the included studies. Low, moderate, and high heterogeneity was considered for levels of I^2^ values of 25–49%, 50–74%, and above 75%, respectively [17]. We applied a random effects model, while if the I^2^ statistic was lower than 50%, we applied a fixed-effect model to obtain pooled HR and 95% CI. The graphical description of the statistical results was illustrated with a Forest plot.

## 3. Results

### 3.1. Study Selection

**Figure 1:**
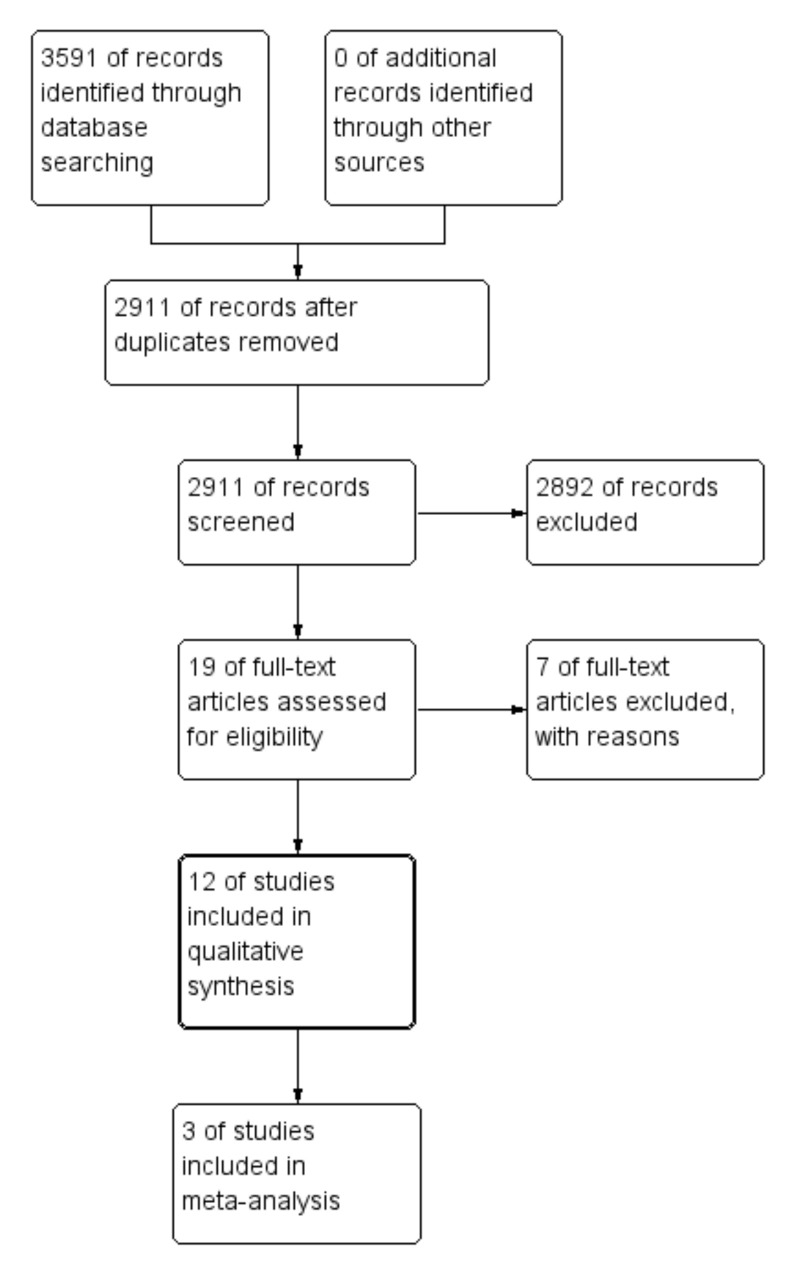
Flowchart of study selection.

After the literature search, 2911 relevant non-duplicated records were identified; 2892 of them were excluded based on the title or the abstract because they covered a variety of irrelevant topics. Finally, 12 studies, published between 2012 and 2021, matched the selection criteria and were included in the quality analysis, as shown in Figure 1 [14,18,19,20,21,22,23,24,25,26,27,28]. The authors of potentially eligible studies with minor missing or incomplete data were directly contacted and invited to contribute additional information and data.

### 3.2. Study Characteristics and Patients Characteristics

Twelve studies, all published between 2012 and 2021, matched the inclusion criteria and have been included in the qualitative analysis [14,18,19,20,21,22,23,24,25,26,27,28]. Five studies were prospective studies and seven were retrospective studies. The characteristics of the included studies are shown in Table 1.

All studies had a quality score ≥6, assessed using the Newcastle–Ottawa score. All the studies reported an accurate description of IC and CTR. Five studies did not report the safety of and tolerance to chemotherapy [14,18,20,26,27]. The analysis of study quality has been summarized in Table 2.

Patient characteristics are shown in Table 3. A total of 1080 patients with PDAC who underwent IC + CRT were analyzed. In two articles, patients had a diagnosis of resectable I PDAC [18,26]. In other articles, patients were classified as BR (N 372, 34%) or LA (N 477, 44%). The resectability status and stage were determined according to NCCN guidelines in all selected articles [29]. Gemcitabine alone or combined was the main administered regimen for IC (N 620, 57%). FOLFIRINOX was the second-most diffused regimen (N 490, 45%).

### 3.3. Response and Tolerance to TNT

The percentage of patients who completed IC varied from 37% to 100%. Grade 3 or greater of toxicity during IC was observed in 14–51% of patients. Disease progression during IC ranged from 3% to 25%. Based on available data in 27 (10%) LA and 17 (13%) BR patients, disease progression during IC was observed. A total of 898 (91%) received CRT after IC. Capecitabine or Gemcitabine were the most commons regimens combined with radiotherapy. In six studies, radiotherapy was administered with a radiation dose of 50.4 Gy in 28 fractions combined with Capecitabine or Gemcitabine. Disease progression during CRT was observed from 6% to 22% of subjects. A total of 607 (62%) patients underwent curative surgery after IC + CRT. Based on the available data, respectively, 77 (61%) BR patients and 124 (43%) LA patients underwent surgery after completion of IC + CRT. A total of 107 (10%) patients underwent only surgical exploration after IC + CRT.

### 3.4. Pathological, Surgical and Survival Outcomes

As shown in Table 4, pathological complete response was observed in 36 (5%) patients. The percentage of regional lymph node metastases varied from 20% to 56%. R0 resection rates varied from 39.2 to 100%. Major complications after surgery, classified as Clavien Dindo ≥ 3b, were observed in 12.5–56% of patients. Median DFS varied from 14.8 to 48.6 months. Median OS varied from 10.8 to 51.5 months.

In Table 5, the main pathological and survival outcomes in patients undergoing curative surgery after completion of TNT were reported, according to stratification by the initial stage. According to the available data, regional lymph node metastases were found in 56% of R-, 34% of BR-, and 36% of LA patients. Resection R0 was found in 90% of R-, 68% of BR-, and 66% of LA patients. Survival outcomes were not available for R patients.

**Figure 2:**
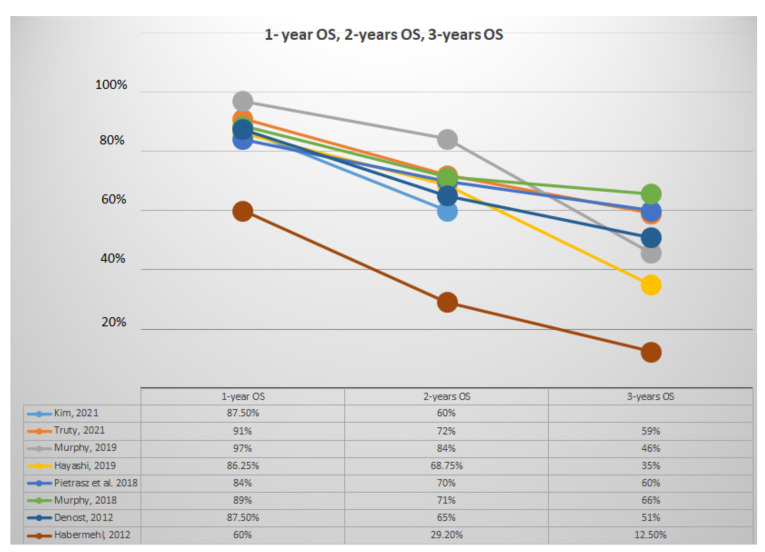
Representative analysis of 1-, 2-, and 3-year OS.

Figure 2 summarized values of 1-year, 2-year, and 3-year OS. Articles with patients with diagnosis of LA PDAC had 1-year OS varying from 60% to 72%, 2-year OS from 29.2% to 48%, and 3-year OS from 12.5% to 15% [21,28]. Articles with patients with a diagnosis of BR PDAC had a 1-year OS varying from 68.6% to 71%, and 2-year OS from 28% to 35.3% [20,22].

Articles with FOLFORINOX as the main regimen had a 1-year OS varying from 84% to 97%, 2-year OS from 70% to 84%, and 3-year OS from 46% to 66% [14,21,22]. Articles with Gemcitabine as the main regimen used had a 1-year OS varying from 60% to 87%, 2-year OS from 29% to 68.75%, and 3-year OS from 12.5% to 51% [20,27,28].

**Figure 3:**
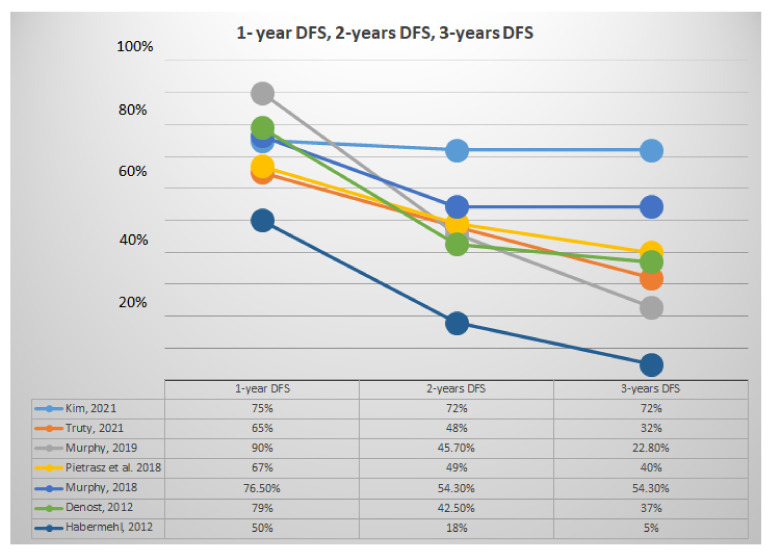
Representative analysis of 1-, 2-, and 3-year DFS.

Figure 3 summarized values of 1-, 2-, and 3-year DFS. The 1-, 2-, and 3-year DFS varied from 50% to 90%, 18% to 72%, and 5% to 72%, respectively. Articles with patients with a diagnosis of LA PDAC had 1-year DFS varying from 50% to 90%, 2-year DFS from 18% to 45.7%, and 3-year DFS from 5% to 37% [21,27,28].

### 3.5. Meta-Analysis: Pathological and Survival outcomes

Three studies described comparable patient groups in terms of pathological outcomes and two studies in terms of survival outcomes (Table 6); thus, a metanalysis was attempted [14,18,20,24].

**Figure 4:**
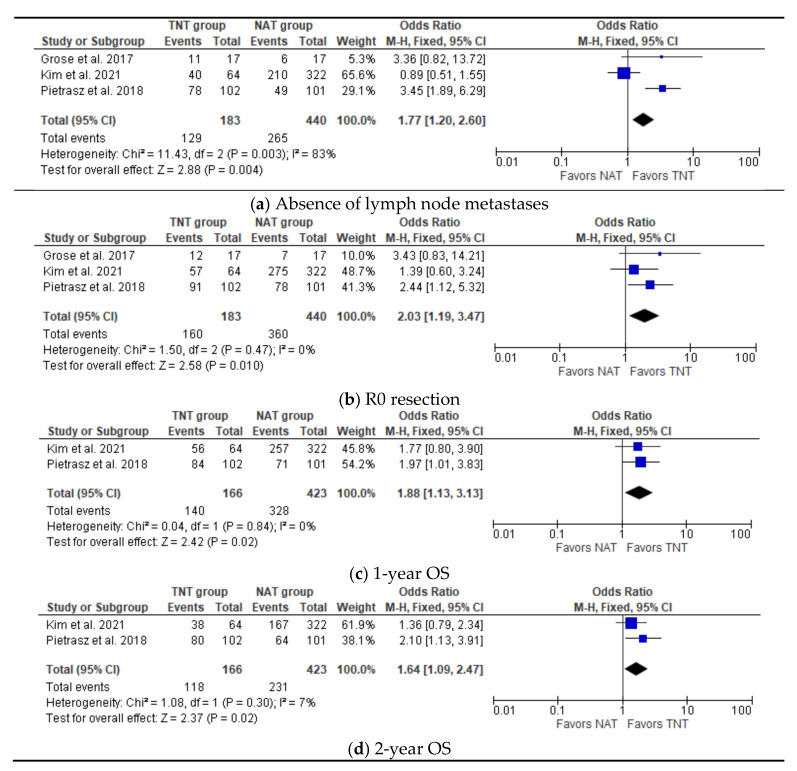
Meta-analysis of study on pathological and survival outcomes. (**a**) Forest plot of absence of lymph node metastases (**b**) Forest plot of R0 resection (**c**) Forest plot of 1-year OS (**d**) Forest plot of 2-year OS.

All three studies investigating patients with PDAC undergoing TNT followed by surgery in comparison with patients undergoing surgery after NAT assessed an intervention group versus a control group. A total of 623 patients have been evaluated, of which 183 underwent intention-to-treat surgery after TNT and 440 underwent intention-to-treat surgery after NAT. As shown in Figure 4, three studies reported data on lymph node metastases and radicality, with a significant benefit for TNT groups (OR 1.77, 95% CI 1.2–2.60, *p* = 0.004 and OR 2.03, 95% CI 1.19–2.60, *p* = 0.01, respectively) [14,18,24]. Furthermore, two studies reported data of 1-year OS and 2-year OS with a significant benefit in favor of the TNT group (OR 1.88, CI 1.13–3.13, *p* = 0.02 and OR 1.64, CI 1.09–2.47, *p* = 0.02) [14,18].

## 4. Discussion

PDAC remains one of the most lethal malignancies, with an overall 5-year survival rate of approximately 5% [30,31]. Unfortunately, only 15–20% of PDAC patients are diagnosed early enough to be resectable and about 50% of them are diagnosed at a metastatic stage [6,32,33].

The improvement in overall survival with adjuvant systemic chemotherapy with 5-fluorouracil plus folinic acid (ESPAC-1 trial) or gemcitabine plus capecitabine (ESPAC-4 trial) has shown a further step change in survival after resection for PDAC [34,35]. Consequently, surgical resection followed by adjuvant chemotherapy is currently considered the best chance of curative treatment and long-term survival for patients with PDAC. Unfortunately, patients who underwent curative surgery were not always eligible and fit enough for adjuvant chemotherapy [13].

In the last years, NAT in PDAC has received both approval and criticism. On the one hand, its benefits are evident, such as the downstaging of large tumors to allow margin negative resections, better patient selection for resection by revealing biological aggressiveness, and the enablement of further observation of indeterminate extrapancreatic lesions prior to resection; on the other hand, the main debate is about the real prognostic advantage and the risk of losing the opportunity of surgery in some patients [9,10,11,12,36]. Recently, TNT has been taking hold in PDAC, based upon excellent results in other types of cancers [37]. IC before CRT may give the theoretical and potential chance to eradicate distant micrometastases at an early stage in the evolution of the disease. Tumor shrinkage after systemic chemotherapy potentially allows improved tumor vascularity that has the consequence of higher intratumoral levels of cytotoxic drugs and higher tumoral sensitivity to CRT [38]. Nevertheless, IC can potentially delay a surgical treatment or select radio-resistant clones, allowing distant seeding and reducing compliance to CRT [39].

Our review showed that the main regimens utilized for IC were Gemcitabine-based and FOLFIRINOX. Despite the increasing interest in TNT for PDAC, an optimal chemotherapy protocol and the proper regimen for IC remains to be established. Patients who completed IC varied from 53.3% to 100% in the Gemcitabine group and from 50.8% to 80% in the FOLFIRINOX group. One of the main criticisms directed to IC was the potential toxicity that could result in missing the surgical intent. This is a controversial issue: as underlined by Ioka et al., a regimen with IC followed by CRT was less toxic than a regimen with only CRT, and patients lost during IC are more likely due to disease progression than toxicity [36]. As shown in Table 3, this concept is well observed in the Gemcitabine regimens where the toxicity varied from 2% to 10% and disease progression during IC varied from 11% to 30%. Differently, the FOLFIRINOX regimens showed more toxicity (10.8% to 51%) but less disease progression (5% to 16%). Consequently, the drop-out rate during IC is largely due to disease progression. As suggested by Abbott et al., these patients present aggressive tumor biology, prone to rapid progression in distant sites, reducing the potential survival benefit of surgery [26]. The benefit of selecting in- (and out-) patients who will (or will not) benefit from CRT should be seen as an advantage of IC [40]. Furthermore, the dropout rate during IC still remains lower than the 24–50% of patients undergoing upfront surgery, who are ineligible to receive adjuvant chemotherapy [41].

The role of CRT in PDAC finds its main rationale in the high local aggressiveness of the disease with a high risk of local recurrence after surgery and high rates of involvement of retroperitoneal margins [11]. Surgical radicality is one of the most important prognostic factors in PDAC [39,40]. In resectable PDAC undergone upfront surgery, Ryan et al., found a 40% to 70% chance of R1 resection [32]. Surgical radicality is even more challenging in certain categories of patients with PDAC, such as LA and BR, mostly represented in our review. In the literature, the role of CRT has long been investigated, especially in its adjuvant setting, where its role is controversial [35,42,43].

As shown in Table 3, patients who received CRT after IC varied from 38.3% to 100%. In prospective studies, this percentage varied from 78% to 95,6%. Despite expected problems of toxicity or disease progression, the majority of patients enrolled for TNT were eligible for CRT. The variability of CRT regimens was also reported: the majority of authors used the same radiotherapy dose of 50.4 Gy given in 28 fractions, while the most frequent concurrent chemotherapy regimens were Gemcitabine or Capecitabine. As underlined by Mukherjee, Gemcitabine is a more potent radiosensitizer than Capecitabine; however, the systemic effect of concomitant chemotherapy during radiotherapy seems to account for the difference [44].

Patients who completed TNT and underwent surgery with curative intent varied from 26% to 100%. Within this variability of drop-out rate, we found both patients who could not benefit from surgical treatment and patients who underwent surgical treatment without completing TNT. Unfortunately, articles do not quantify the latter category of patients.

The uncertainty of not reaching surgical resection is one of the debated topics in neoadjuvant therapy; our review showed that patients who underwent only exploratory laparotomy varied from 2% to 28%, with the highest percentages among patients with a diagnosis of LA PDAC [21,25,28]. The resectability rates after TNT, shown in Table 3 and Table 5, tended to be similar or higher than those reported in the literature after neoadjuvant therapy, also considering authors such as Palmer et al., who showed a resectability rate of 54% in patients with resectable (R) PDAC after NAT; and Versteijne et al., who reported a resection rate for patients with R and BR pancreatic cancer of 67% and 65%, respectively [45].

The wide heterogeneity and range of data reported in selected articles are certainly due to different nature of studies; in particular, retrospective articles showed better surgical and oncological outcomes, with resection rates up to 100%; we can speculate that better results could depend on implicit selection bias with the recruitment of patients who are fitter for TNT and surgery. On the contrary, prospective studies showed higher percentages of drop-out and worse surgical and oncological outcomes. Furthermore, the median OS varied widely across the articles, as shown in Table 4, ranging from 10,8 to 51,1 months: this can be explained firstly by the different group of patients at diagnosis, as confirmed by data reported in Table 5 with patients stratified by the initial stage of disease. Nevertheless, initial chemotherapy regimens seem to play an equally determinant role in surgical and oncological outcomes. In Figure 2 and Figure 3, we showed survival trends in the first 3 years after surgery: both OS and DFS seemed to be more influenced by the type of IC regimen administered than by the initial stage and completion of CRT. In particular, articles with FOLFIRINOX as the predominant chemotherapy regimen seemed to have better survival outcomes [14,18,19,21,22]. This finding is consistent with the observation that PDAC should be considered a systemic disease, which could benefit from an aggressive systemic chemotherapy. Nonetheless, as shown in Table 6 and Figure 4, articles reporting comparison among patients (10.5% R, 65% BR, 24.5% LA) treated with TNT and NAT showed better pathological and oncological outcomes in favor of the TNT group, even though the results could be affected by IC regimen.

## 5. Conclusions

To our knowledge, this is the first systematic review investigating the role of TNT and surgery with curative intent in PDAC, with a comparison to NAT without CRT. Several limitations need to be considered when interpreting our data: the retrospective nature of some articles, the wide heterogeneity of IC and CRT regimens, and different groups of patients starting TNT, which make it difficult to obtain homogeneous and easily comparable results.

However, despite its limitations, this review suggests that TNT can be considered a good therapeutic pathway for patients, especially BR- and LA PDAC ones, who may benefit from surgical treatment with curative intent, with good survival and acceptable morbidity. Moreover, current evidence demonstrates the potential superiority of TNT compared to NAT without CRT in oncological and pathological outcomes, in particular in patients with doubtful resectability at the end of neoadjuvant treatment.

Prospective randomized trials are certainly needed to verify whether TNT can be considered a standard of care in patients with PDAC, to determine the best IC and CRT regimen, and to identify which patients may benefit the most from this therapeutic approach.

## Figures and Tables

**Table 1:** Characteristics of studies analyzed.

References	Publication Year	Centre	Country	Study Design	Inclusion Period	No. of Patients
Kim et al. [18]	2021	Medical College of Wisconsin	USA	Retrospective	2009–2019	89
Truty et al. [19]	2021	Mayo Clinical College of Medicine	USA	Retrospective	2010–2017	254
Hayashi et al. [20]	2019	Hokkaido Pancreas Study Group (HOPS)	JAPAN	Prospective	2013–2015	45
Murphy et al. [21]	2019	Massachusetts General Hospital	USA	Prospective	2013–2018	49
Murphy et al. [22]	2018	Massachusetts General Hospital	USA	Prospective	2012–2016	48
Takahashi et al. [23]	2018	Osaka International Cancer Institute	JAPAN	Prospective	Not specified	38
Pietrasz et al. [14]	2018	Paul Brousse Hospital	FRANCE	Retrospective	2010–2015	203
Grose et al. [24]	2017	Beatson West of Scotland Cancer Centre	UK	Retrospective	2012–2015	85
Fiore et al. [25]	2017	Campus Bio-Medico University Rome	ITALY	Prospective	2012–2015	41
Abbott et al. [26]	2013	University of Cincinnati School of Medicine	USA	Retrospective	Not specified	164
Denost et al. [27]	2012	University Hospital Centre (CHU) Bordeaux	FRANCE	Retrospective	2004–2009	111
Habermehl et al. [28]	2012	University Hospital of Heidelberg	GERMANY	Retrospective	2001–2010	215

**Table 2:** Level of evidence and quality assessment of the selected studies.

References	No. of Patients	Accurate Description of IC	Accurate Description of CRT	Accurate Description of Safety and Tolerance to IC + CRT	Accurate Description of Surgical Procedure	Newcastle–Ottawa Score			
						Selection	Comparability	Outcome	Score
Kim et al., 2021 [18]	89	Yes	Yes	No	Yes	****	*	***	8
Truty et al., 2021 [19]	254	Yes	Yes	Yes	Yes	***	-	***	6
Hayashi et al., 2019 [20]	45	Yes	Yes	No	Yes	***	-	***	6
Murphy et al., 2019 [21]	49	Yes	Yes	Yes	Yes	***	-	***	6
Murphy et al., 2018 [22]	48	Yes	Yes	Yes	Yes	***	-	***	6
Takahashi et al., 2018 [23]	38	Yes	Yes	Yes	Yes	***	-	***	6
Pietrasz et al. [14]	203	Yes	Yes	No	Yes	****	*	***	8
Grose et al., 2017 [24]	85	Yes	Yes	Yes	No	****	*	***	8
Fiore et al., 2017 [25]	41	Yes	Yes	Yes	No	****	*	***	8
Abbott et al., 2013 [26]	164	Yes	Yes	No	No	****	*	***	8
Denost et al., 2012 [27]	111	Yes	Yes	No	Yes	****	*	***	8
Habermehl et al., 2012 [28]	215	Yes	Yes	Yes	No	***	-	***	6

Newcastle–Ottawa Quality Assessment Scale (*: the study met the criteria for a domain of the Newcastle–Ottawa Scale, each * represents if individual criterion within the subsection was fulfilled; -: the criteria were not met). Newcastle-Ottava Scale for Case-Control studies (Selection: 1. Adequacy of case definition, 2. Representativeness of the cases, 3. Selection of controls, 4. Definition of Controls; Comparability: 1. Comparability of cases and controls on the basis of the design; Exposure: 1. Ascertainment of exposure, 2. Same method of ascertainment for cases and con-trols, 3. Non-Response rate) and Cohort studies (Selection: 1. Representativeness of the exposed cohort, 2. Selection of the non-exposed cohort, 3. Ascertainment of exposure, 4. Demonstration that outcome of interest was not present at start of study; Compa-rability: 1. Comparability of cohorts on the basis of the design or analysis; Outcome: 1. Assessment of outcome, 2. Was follow-up long enough for outcomes to occur, 3. Adequacy of follow up of cohorts).

**Table 3:** Characteristic of patients who underwent IC, CRT, and surgery.

			Induction Chemotherapy (IC)					Patients Received CRT after or before IC, N (%)	ChemoRadioTherapy (CRT)			Surgery after IC + CRT	
Reference, Year	Number of Patients, N	Classification of Tumor, N	Regimen, N (%)	Cycles, N	Completion of IC ***, N (%)	Grade 3 or Greater Toxicity ****, N (%)	PD during IC, N (%)		Regimen	Radiotherapy Dose	PD during CRT, N (%)	Patients Undergoing Pancreatic Resection after IC + CRT, N (%)	Patients Undergoing only Surgical Exploration, N (%)
Kim et al., 2021 [18]	89	R 22, BR 67	FOLFIRINOX 66 (74), Gem/Nab 17 (19)	8 *	64 (72)	Ns	19 (21)	86 (97)	Cap or Gem	50.4 Gy in 28 fractions	19 (22)	64 (72)	Ns
Truty et al., 2021 [19]	194	LA 71, BR 123	FOLFIRINOX 165 (85) or Gem/Nab 65 (34)	6 **	71 (37)	32 (14)	25(10)	194 (100)	Cap or 5FU or Gem	50.4 Gy in 28 fractions	Ns	194 (100)	0
Hayashi et al., 2019 [20]	45	BR 45	Gem 45 (100)	8 *	24 (53,3)	Ns	9 (25)	43 (95,6)	S-1	50.4 Gy in 28 fractions	4 (8)	24 (53,3)	1 (2)
Murphy et al., 2019 [21]	49	LA 49	FOLFIRINOX 49 (100)	8 *	39 (80)	25 (51)	5 (10)	45 (92)	Cap or 5FU	50.4 Gy in 28 fractions or 25 GyE in 5GyE	3 (6)	34 (69)	8 (16)
Murphy et al., 2018 [22]	43	BR 43	FOLFIRINOX 43 (100)	8 *	34 (79)	9 (19)	2 (5)	39 (90)	Cap or 5FU	50.4 Gy in 28 fractions or 25 GyE in 5GyE	3 (6)	29 (67)	4 (9)
Takahashi et al., 2018 [23]	38	BR 38	Gem/Nab 38 (100)	2	30 (78)	1 (2)	6 (15)	30 (78)	Gem/Nab	60 Gy in 25 fractions	5 (17)	24 (80)	Ns
Pietrasz et al. 2018 [14]	102	BR 49, LA 53	FOLFIRINOX 102 (100)	6 *	24 (23,5)	Ns	Ns	102 (100)	Cap or Gem	49 to 59 Gy in 30 fractions	Ns	102 (100)	0
Grose et al., 2017 [24]	85	BR 45, LA 40	FOLFIRINOX 65 (76)	6 *	33 (50,8)	7 (10,8)	16 (24,6)	33 (38,3)	Cap	50.4 Gy in 28 fractions	Ns	17 (51)	2 (6)
			Gem-Cap 20 (24)	3 *	14 (70)	3 (10)	6 (30)						
Fiore et al., 2017 [25]	34	LA 27, BR7	Gem and Oxaliplatin 34 (100)	4 *	34 (100)	3 (8)	5 (14,7)	27 (79)	Gem	54 Gy (BRPC) or 59,4 Gy (LA) in 28 fractions	5 (18,5)	15 (55)	4 (14)
Abbott et al., 2013 [26]	164	R 164	Gem 164 (100)	4 *	164 (100)	Ns	Ns	164 (100)	Gem	30 Gy in 10 fractions	18 (10)	116 (71)	12 (7)
Denost et al., 2012 [27]	39	LA 39	Gem or GEMCIS 39 (100)	Ns	Ns	Ns	Ns	39 (100)	5FU	45 Gy in 25 fractions	Ns	39 (100)	0
Habermehl et al., 2012 [28]	198	LA 198	Gem 198 (100)	Ns	Ns	Ns	22 (11)	198 (100)	Gem	52,2 Gy (Intraoperative radiotherapy 15 Gy in 26 patients)	Ns	51 (26)	53 (28)

* Expected cycles, ** median number of cycles performed, *** patients who completed IC or made >8 cycles, **** Criteria for Adverse Events, version 3.0. IC: induction chemotherapy; PD: disease progression; CRT: chemoradiotherapy; LA: locally advanced; BR: borderline resectable; R: resectable; Gem-Nab: Gemcitabine-Nab-paclitaxel, Gem Gemcitabine, Cap Capecitabine; 5-FU: 5 Fluorouracil; FOLFIRINOX: oxaliplatin, irinotecan, fluorouracil, and leucovorin; Ns: not specified.

**Table 4:** Pathological characteristics and short-term outcomes of patients who underwent surgery after IC + CRT.

			Pathological Outcomes			Surgical Outcomes		Long-Term Outcomes	
Reference	Patients Undergone Surgery after IC + CRT, N (%)	Type of Surgery, N (%)	Pathological Complete Response, N (%)	Regional Lymph Node Metastases, N (%)	Resection R0, N (%)	Major Complications after Surgery, N (%)	90 Day Mortality, N (%)	DFS, Median (Months)	OS, Median (Months)
Kim et al., 2021 [18]	64 (72)	PDC 53 (83) DP 6 (9) TP 5 (8)	5 (8)	25 (34)	57 (89)	36 (56)	Ns	Ns	Ns
Truty et al., 2021 [19]	194 (100)	PDC 122 (63) TP 25 (13)	0	39 (20)	183 (94)	69 (36)	13 (6,7)	23,5	51,1
Hayashi et al., 2019 [20]	24 (53,3)	PDC 19 DP 4 TP 1	0	6 (25)	23 (95,8)	6 (25)	Ns	14,8	27,9
Murphy et al., 2019 [21]	34 (69)	Ns	3 (9)	9 (26)	30 (88)	Ns	Ns	21,3	33
Murphy et al., 2018 [22]	29 (67)	Ns	0	20 (38)	29 (100)	Ns	Ns	48,6	Ns
Takahashi et al., 2018 [23]	24 (80)	PDC 12 DP 12	3 (12)	Ns	23 (96)	3 (12,5)	0	Ns	Ns
Pietrasz et al. 2018 [14]	102 (100)	Ns	22 (10,8)	24 (23,5)	169 (83,3)	Ns	Ns	17.7	47.9
Grose et al., 2017 [24]	17 (51)	Ns	3 (17)	6 (35)	12 (70,6)	Ns	Ns	Ns	Ns
Fiore et al., 2017 [25]	15 (55)	Ns	0	Ns	15 (100)	Ns	0	35,2	37,6
Abbott et al., 2013 [26]	116 (71)	Ns	Ns	65 (56)	104 (90)	27 (23)	1 (1)	Ns	Ns
Denost et al., 2012 [27]	39 (100)	PDC 39 (100)	Ns	16 (41)	33 (84,6)	12 (30)	Ns	Ns	Ns
Habermehl et al., 2012 [28]	51 (26)	Ns	Ns	Ns	20 (39,2)	Ns	Ns	10,8	10,8

PD: pancreaticoduodenectomy; DP: distal pancreatectomy; TP: total pancreatectomy; Ns: not specified; DFS: disease-free survival; OS: overall survival.

**Table 5:** Pathological characteristics and survival outcomes subdivided for diagnostic classes of patients who underwent surgery after TNT.

Classification of Patients	Patients Undergone Surgery after TNT	Regional Lymph Node Metastases, N (%)	Resection R0, N (%)	1-Year OS	2-Years OS	1-Year DFS	2-Years DFS
**Resectable**	116	65 (56%)	104 (90%)	ns	ns	ns	ns
**Borderline resectable**	94	32 (34%)	64 (68%)	47 (88%)	36 (67%)	22 (76,5%)	15 (54%)
**Locally advanced**	124	45 (36%)	83 (66%)	97 (78%)	68 (54%)	86 (69%)	40 (32%)

ns: not specified.

**Table 6:** Comparison between patients with PDAC who underwent Surgery after TNT versus Surgery after NAT.

			Pathological Outcomes						Surgical Outcomes		Long-Term Outcomes	
Reference	Patients Undergone Surgery after IC + CRT, N (%)	Patients Undergone Surgery after NAT	Pathological Complete Response, N (%)		Regional Lymph Node Metastases, N (%)		Resection R0, N (%)		Major Complications after Surgery, N (%)		1-Year, 2-Year, 3-Year OS, Percentage	
			TNT	NAT	TNT	NAT	TNT	NAT	TNT	NAT	TNT	NAT
Kim et al., 2021 [18]	64	322	5 (8)	13 (4)	25 (34)	122 (38)	57 (89)	275 (85)	36 (56)	189 (59)	87,5%, 60%, Ns	80%, 52%, 37%
Grose et al.,2017 [24]	17	17	3 (17)	Ns	6 (35)	11 (64)	12 (70,6)	7 (47,6)	Ns	Ns	Ns	Ns
Pietrasz et al. 2018 [14]	102	101	17 (16,7)	5 (5)	24 (23,5)	52 (51,5)	91 (89,2)	78 (76,3)	Ns	Ns	84%, 70%, 60%	80%, 63%, 44%

Ns: not specified.

## Data Availability

Data analyzed is contained within the article.
